# To His Excellency the Honourable Frederic North, Governor of Ceylon, &c. &c. &c.

**Published:** 1803-06-01

**Authors:** Thomas Christie

**Affiliations:** Medical Superintendent General. Columbo


*Columbo,*

*September* 1, 1802.

T. CHRISTIE.

---

*To His Excellency the Honourable* FREDERIC NORTH,
*Governor of Ceylon, &c. &c. &c.*

SIR,

THE Vaccine disease, that safe and certain antidote against the Small-pox, being at length successfully introduced into Ceylon, I consider the Small-pox Hospitals as now unnecessary, and therefore beg leave to recommend to your Excellency, that the further admission of patients into these hospitals be prohibited; and that as soon as the cases at present under treatment are terminated, the hospitals be shut up, and the establishments suppressed.

The Cow-pox is a disease of so mild and innocent a nature as not to require any particular confinement or treatment; but as it is of the utmost importance that the virus should be kept up in an uncontaminated state, and that the Vaccine disease should be propagated throughout the island with as much certainty and expedition as possible, I think the present establishment of Medical Superintendents and Overseers may, with great advantage, be employed for this purpose, as well as for affording medical aid to the natives in case of accidents or epidemics, superintending quarantines, and watching over and reporting every circumstance connected with the health of the people

The care of keeping up the Vaccine contagion, ought, I think to be particularly entrusted to the medical superintendents intendents at each station, who will see that all the overseers in their districts are well instructed in the practice of Vaccine Inoculation, and that they are actively employed in disseminating this most beneficial practice.

The Medical Overseers at present receive ninety rix-dollars when employed in actual service in an infected village; and I would recommend that the same allowance should be continued to them when actually employed in propagating the Vaccine disease in any village at ten miles distance from their usual residence: probably two months in the year would be sufficient for inoculating all the children in the more remote parts of the country, when the different overseers might make the circuits of all the villages in their districts, during which period they would be allowed to draw ninety rix-dollars per mensem; and at all other times they would receive the regular salary of thirty rix-dollars monthly.

Should these proposals meet your Excellency's approbation, I shall immediately write to the different Medical Superintendents, informing them of your Excellency's commands, and at the same time forward their instructions with respect to reports of their practice, &c. &c.

I have the honour to be, &c.

*Columbo,* THOMAS CHRISTIE,

*Sept.* 4, 1802. Medical Superintendent General.

